# Health literacy and primary health care use of ethnic minorities in the Netherlands

**DOI:** 10.1186/s12913-017-2276-2

**Published:** 2017-05-15

**Authors:** Marieke van der Gaag, Iris van der Heide, Peter M. M. Spreeuwenberg, Anne E. M. Brabers, Jany J. D. J. M. Rademakers

**Affiliations:** 1 NIVEL – Netherlands Institute for Health Services Research, PO Box 1568, 3500 BN Utrecht, Netherlands; 2 Department of Family Medicine, Maastricht University, CAPHRI Care and Public Health Research Institute, Maastricht, Netherlands

**Keywords:** Health literacy, Ethnic minorities, General practitioner, Primary care

## Abstract

**Background:**

In the Netherlands, ethnic minority populations visit their general practitioner (GP) more often than the indigenous population. An explanation for this association is lacking. Recently, health literacy is suggested as a possible explaining mechanism. Internationally, associations between health literacy and health care use, and between ethnicity and health literacy have been studied separately, but, so far, have not been linked to each other. In the Netherlands, some expectations have been expressed with regard to supposed low health literacy of ethnic minority groups, however, no empirical study has been done so far. The objectives of this study are therefore to acquire insight into the level of health literacy of ethnic minorities in the Netherlands and to examine whether the relationship between ethnicity and health care use can be (partly) explained by health literacy.

**Methods:**

A questionnaire was sent to a sample of 2.116 members of the Dutch Health Care Consumer Panel (response rate 46%, 89 respondents of non-western origin). Health literacy was measured with the Health Literacy Questionnaire (HLQ) which covers nine different domains. The health literacy levels of ethnic minority groups were compared to the indigenous population. A negative binomial regression model was used to estimate the association between ethnicity and GP visits. To examine whether health literacy is an explaining factor in this association, health literacy and interaction terms of health literacy and ethnicity were added into the model.

**Results:**

Differences in levels of health literacy were only found between the Turkish population and the indigenous Dutch population. This study also found an association between ethnicity and GP visits. Ethnic minorities visit their GP 33% more often than the indigenous population. Three domains of the HLQ (the ability to navigate the health care system, the ability to find information and to read and understand health information) partly explained the association between ethnicity and GP visits.

**Conclusions:**

In general, there are no differences in health literacy between most of the ethnic minority groups in the Netherlands and the indigenous Dutch population. Only the Turkish population scored significantly lower on several health literacy domains. Some domains of health literacy do explain the association between ethnicity and higher frequency of GP visits. Further research is recommended to understand the pathways through which health literacy impacts health care use.

## Background

Internationally, several studies report differences in general practitioner (GP) visits between adults from ethnic minorities and from indigenous populations. However, these findings are not consistent [19]. In the Netherlands, ethnic minorities visit the GP relatively more often [32], whereas in other European countries ethnic minorities have less GP visits [10, 14]. Some studies have explored possible underlying mechanisms that could explain the association between ethnicity and health care use. These studies report that the association between ethnicity and health care use can be partly explained by education, knowledge, language proficiencies and access to care [2, 17, 36].

In the Dutch health care system, the GP plays an important role as he or she is the gatekeeper to specialized medical care [23, 29]. Ethnic minority populations in the Netherlands visit the GP more often compared to the indigenous population [29, 32]. Individuals of the four largest ethnic minority groups in the Netherlands (i.e., people from Morocco, Turkey, Surinam and the Netherlands Antilles) approximately have 1.5 times as much contact with their GP compared to the indigenous Dutch population [33]. However, an explanation why ethnic minority populations in the Netherlands have more contact with their GP is lacking [33].

A factor that has internationally gained attention in recent years and that is considered to be a possible explaining mechanism in the association between ethnicity and primary care use, is health literacy [4, 16]. An association between health literacy and health care use has been found in earlier studies. People with low health literacy use more health care services, including GP visits, hospitalization and emergency care [5, 34].

Several definitions of health literacy exist [24, 27]. In the present study, the definition of the World Health Organization (WHO) is used: “The cognitive and social skills which determine the motivation and ability of individuals to gain access to, understand and use information in ways which promote and maintain good health” [24]. Having lower health literacy has been associated with poorer health outcomes and contributes to health disparities [4, 5, 9, 15]. According to the WHO, in fact, health literacy predicts health outcomes better than age, educational level, income, job and cultural background [13].

Often health literacy is conceptualized as having basic reading and writing skills (i.e. functional health literacy). More advanced definitions discern different types of skills, e.g. functional, communicative and critical skills [20], or different levels, e.g. access, understand, appraise and apply health information [27]. Due to differences in the conceptualization of health literacy, a wide range of measurement instruments has been designed. The first health literacy measurement methods focused on functional health literacy only [1, 22, 28]. In recent research, more extended tools, like the Health Activities Literacy Scale (HALS), the European Health Literacy Survey (HLS-EU) and the Health Literacy Questionnaire (HLQ), have been developed and validated [21, 26, 35]. In this study, we used the HLQ, because it measures health literacy in a comprehensive way. Similar to the definition of the WHO, this multidimensional instrument distinguishes both cognitive, psychosocial and social aspects, that each may affect health behaviour, such as health care use, in a different way.

Whereas it is known that there is a link between low health literacy and health care use [5, 34], it is not known what the association is between ethnicity and health literacy in the Netherlands. In Canada and the USA, studies found that the level of health literacy is lower in ethnic minority groups compared to the indigenous population [11, 18, 25]. Although some expectations have been expressed with regard to supposed low health literacy of ethnic minority groups in the Netherlands [12], no empirical study has been done yet. The first aim of our study is therefore to acquire insight in the level of health literacy of ethnic minorities in the Dutch population.

Internationally, the associations between health literacy and health care use, and between ethnicity and health literacy have been studied separately, but so far, have not been linked to each other. This is the second aim of our study. Only Ackermann Rau and colleagues have done a study on health literacy, ethnicity and health care use in Switzerland. However, in this study health care use was defined as knowledge when to seek help [2]. Their results showed that migrants with relatively lower health literacy misinterpreted minor symptoms more often, and therefore potentially overused primary care. Only migrant groups were compared with each other, as the indigenous population was not included in the sample [2].

In summary, this study aims to: a) acquire insight into the level of health literacy of ethnic minorities in the Netherlands; b) examine whether the relationship between ethnicity and health care use is (partly) explained by health literacy. To perform this study, the following research questions were formulated:

1. ‘Do health literacy skills of ethnic minority groups and of the indigenous population in the Netherlands differ?’
2. ‘Do ethnic minority groups and the indigenous population in the Netherlands differ with respect to GP visits?’
3. ‘Is health literacy an explanatory mechanism in the association between ethnicity and GP visits?’

## Methods

### The Dutch health care consumer panel

The data that were used for this cross-sectional study were obtained from the NIVEL Dutch Health Care Consumer Panel [6]. This panel provides information about opinions and knowledge about health care, and expectations and experiences with health care. At the moment of this study (May 2015), the Consumer Panel consisted of approximately 12.000 people aged 18 years and older. Background characteristics from all panel members, such as ethnicity, gender, age and highest level of education completed, were assessed at the start of the panel membership. Each year, approximately eight surveys are conducted. Each individual panel member receives a questionnaire about three times a year and can resign from the panel at any time. There is no possibility of people signing up for the panel on their own initiative. The Dutch Health Care Consumer Panel is renewed on a regular basis. More details on the recruitment and selection of panel members are reported elsewhere [6]. Data are processed anonymously and the protection of the data collected is registered with the Dutch Data Protection Authority (nr. 1262949). A privacy regulation is available for the Consumer Panel. There is no legal requirement to obtain informed consent nor approval by a medical ethics committee when conducting research through the panel [7].

### Study population

The present study is based on data from 2.116 members of the Dutch Health Care Consumer Panel who received a questionnaire in late May 2015. The sample consisted of all ethnic minorities (both western and non-western) included in the panel (*N* = 1.058) and the same number of members of the indigenous population. The group of the indigenous population was matched to the group of ethnic minorities with respect to gender, age and educational level. Subsequently, 974 responders returned the questionnaire (response rate 46%). In this study, western ethnic minorities were excluded from the final analyses, since the aim was to focus on differences in ethnic background. The ethnic background of western ethnic minorities is overall more comparable to the indigenous population and, therefore, the analyses were performed on the data of 621 responders, of which 89 of non-western origin.

### Variables

#### Health literacy

In the present study, we approached health literacy as a broader concept, including cognitive, motivational and social skills in relation to ethnicity and health care use. To this purpose, we used the Health Literacy Questionnaire (HLQ) developed by Osborne and colleagues [21]. The HLQ measures health literacy with 44 items, divided over nine domains. These domains are: 1) “Feeling understood and supported by health care providers”, 2) “Having sufficient information to manage my health”, 3) “Actively managing my health”, 4) “Social support for health”, 5) “Critical appraisal of health information”, 6) “Ability to actively engage with health care providers”, 7) “Navigating the health care system”, 8) “Ability to find good health information” and 9) “Reading and understanding health information enough to know what to do”. In the first five scales, the respondents were asked: to what extent do you agree with the following statements? The answering options were: 1) strongly disagree, 2) disagree, 3) agree and 4) strongly agree. Examples of the statements are: *“I have enough information to help me deal with my health problems”* and *“I always compare health information from different sources and decide what’s best for me”*. For the last four scales, the respondents were asked how easy or difficult certain tasks are for them at this moment. The answering options were: 1) cannot do, 2) very difficult, 3) quite difficult, 4) quite easy, 5) very easy. Examples of the questioned tasks are: “*Have good discussions about your health with doctors”* and *“Read and understand written health information”*. In our study, the Dutch version of the HLQ is used. This version is translated and validated by Heijmans and colleagues (Heijmans et al., *in preparation)*. The composite reliability of all scales in the Dutch version of the HLQ is >0.74, which is comparable with the original questionnaire [21].

The mean score of the items was used to construct a scale score for each of the nine domains per respondent. If responses to more than two items in a scale were missing, the data of this scale was considered missing. Otherwise scale scores were calculated based on the remaining items. Scale scores were analysed as a continuous outcome.

#### General practitioner visits

Frequency of GP visits was measured with the question: “How often did you consult your GP in 2015?” Consults with a GP included visits at the general practice, visits at home and telephone consultations. Telephone consults concerning drug prescriptions were excluded. The number of GP visits was analysed as a count outcome.

#### Sociodemographics

The following sociodemographics were included: gender, highest level of education completed (categorical), age (continuous) and ethnicity (categorical). Highest level of education completed is categorized as low (primary school of preparatory vocational training), middle (intermediate or advanced general education or intermediate vocational training) and high (high vocational education of university). For the purpose of this study we only selected the indigenous population and non-western ethnic minorities. Someone is defined as non-western migrant if at least one of the parents is born in Turkey, Asia (excluding Japan and Indonesia), Middle or South America or Africa. In the first research question, the ethnic groups have been defined as the indigenous population and the four largest ethnic minority groups, which are: “Turks”, “Moroccans”, “Netherlands Antilleans” and “Surinamese”. In the second and third research question, ethnicity was defined as the indigenous population vs ethnic minorities. In this case, ethnic minorities also included individuals from non-western countries other than specified in the first research question.

### Statistical analyses

To answer the first research question, health literacy profiles were illustrated for each of the four ethnic minority groups and the indigenous population, based on the nine health literacy scales. Multiple Analyses of Covariance (ANCOVAs) in combination with post-hoc analyses were used to examine whether the scores on the nine different health literacy scales of the four ethnic minority groups differed significantly from the indigenous population. The post-hoc analyses were performed with pairwise comparisons and the Tukey Honest Significance Difference (HSD) test [31]. Based on literature, age, level of education and gender were identified as possible confounders. In the final model only age and level of education were included, because age and level of education changed the coefficient of ethnicity with more than 10%, and gender did not..

To answer the second research question a regression analysis was performed. Due to the nature of the data, the count outcome had to be analyzed using a Poisson regression or a negative binomial regression. Since the mean was not equal to the variance of the outcome, it was chosen to perform a negative binomial regression analysis. Both age, level of education and gender were added as a confounder in this model, based on significance *(p < 0.05)*.

Lastly, the health literacy domains where the scores significantly differed in the first research question were put in the negative binomial regression model to examine if health literacy is an explaining variable in the association between health literacy and GP visits. Interaction terms of ethnicity and health literacy were made to examine whether the effect of this association is different for ethnic minorities and the indigenous population. In all analyses, results were considered significant when *p* < 0.05. All analyses were performed using STATA, version 14.0.

## Results

### Sample characteristics

Table 1 shows the sample characteristics. The mean age of the included respondents is 60.6 years (SD = 16.6) and a bit more than half of the respondents are female (51.5%). The sample reports an average of 2.33 GP visits from January until May 2015. With respect to health literacy scores, unadjusted mean scores are higher in the indigenous population compared to the ethnic minorities, except for domain five (see Table 1).

**Table 1:** Sample characteristics

Characteristics	Total (*n* = 621)		Indigenous population (*n* = 532)		Ethnic minorities (*n* = 89)	
	%	*n*	%	*n*	%	*n*
Gender						
Male	48.5	301	48.9	260	46.1	41
Female	51.5	320	51.1	272	53.9	48
Ethnicity						
Turkish					18.0	16
Moroccan					13.5	12
Surinamese					31.5	28
Netherlands Antillean					11.3	10
Other non-western countries					25.9	26
Education						
Low	17.8	109	18.6	99	12.2	10
Middle	49.0	301	49.3	261	47.6	39
High	33.2	204	32.1	171	40.2	33
	Mean (SD)		Mean (SD)		Mean (SD)	
Age	60.64 (16.62)		62.50 (16.04)		49.50 (15.77)	
GP visits	2.33 (2.76)		2.35 (2.84)		2.21 (2.23)	
Health Literacy Scores						
Domain 1 - Provider support	2.85 (0.46)		2.86 (0.44)		2.77 (0.59)	
Domain 2 - Sufficient information	2.85 (0.40)		2.86 (0.39)		2.80 (0.49)	
Domain 3 - Managing health	2.79 (0.43)		2.80 (0.42)		2.77 (0.49)	
Domain 4 - Social support	2.81 (0.46)		2.82 (0.45)		2.71 (0.56)	
Domain 5 – Critical appraisal	2.61 (0.47)		2.60 (0.46)		2.65 (0.55)	
Domain 6 – Active engagement	3.79 (0.61)		3.81 (0.59)		3.67 (0.73)	
Domain 7 – Navigation	3.68 (0.58)		3.71 (0.55)		3.52 (0.72)	
Domain 8 – Finding information	3.79 (0.59)		3.81 (0.57)		3.69 (0.73)	
Domain 9 – Read and understand	3.87 (0.56)		3.88 (0.54)		3.80 (0.70)

**Table 2:** Results of the ANCOVAs and Tukey HSD tests

	ANCOVA		Tukey HSD		
	Unadjusted model *p*	Adjusted model^a^ *p*	Ethnicity	*p*	Adjusted means^a^ (SD)
Domain 1 Provider support	0.117	0.107	Indigenous	ref	2.86 (0.02)
			Turkish	0.315	2.60 (0.12)
			Moroccan	0.766	3.05 (0.14)
			Surinamese	0.715	2.73 (0.09)
			Netherlands Antillean	0.933	2.72 (0.15)
Domain 2 Sufficient information	**0.002**	**0.001**	Indigenous	ref	2.86 (0.02)
			Turkish	**0.006**	2.48 (0.11)
			Moroccan	0.946	2.97 (0.12)
			Surinamese	0.979	2.92 (0.08)
			Netherlands Antillean	0.081	2.50 (0.13)
Domain 3 Managing health	0.207	0.476	Indigenous	ref	2.79 (0.02)
			Turkish	0.509	2.58 (0.12)
			Moroccan	0.998	2.86 (0.15)
			Surinamese	0.947	2.88 (0.09)
			Netherlands Antillean	1.000	2.75 (0.15)
Domain 4 Social support	0.428	0.182	Indigenous	ref	2.83 (0.02)
			Turkish	0.601	2.63 (0.12)
			Moroccan	0.540	2.59 (0.14)
			Surinamese	0.973	2.76 (0.09)
			Netherlands Antillean	0.936	2.68 (0.15)
Domain 5 Critical appraisal	0.063	0.109	Indigenous	ref	2.60 (0.02)
			Turkish	0.869	2.45 (0.13)
			Moroccan	0.301	2.92 (0.15)
			Surinamese	0.861	2.72 (0.10)
			Netherlands Antillean	0.810	2.40 (0.16)
Domain 6 Active engagement	**0.004**	**0.001**	Indigenous	ref	3.81 (0.03)
			Turkish	**<0.001**	3.10 (0.17)
			Moroccan	0.984	3.95 (0.20)
			Surinamese	0.847	3.66 (0.12)
			Netherlands Antillean	0.995	3.70 (0.21)
Domain 7 Navigation	**0.007**	**0.001**	Indigenous	ref	3.72 (0.03)
			Turkish	**0.001**	3.07 (0.16)
			Moroccan	0.999	3.80 (0.20)
			Surinamese	0.765	3.55 (0.12)
			Netherlands Antillean	0.804	3.48 (0.19)
Domain 8 Finding information	**0.019**	**<0.001**	Indigenous	ref	3.82 (0.03)
			Turkish	**<0.001**	3.12 (0.16)
			Moroccan	1.000	3.78 (0.19)
			Surinamese	1.000	3.85 (0.12)
			Netherlands Antillean	0.893	3.61 (0.20)
Domain 9 Read and understand	0.079	**0.009**	Indigenous	ref	3.90 (0.02)
			Turkish	**0.009**	3.40 (0.14)
			Moroccan	0.999	3.97 (0.19)
			Surinamese	0.999	3.94 (0.11)
			Netherlands Antillean	1.000	3.89 (0.18)

Significant results are printed in bold

^**a**^ Adjusted for age, level of education and gender

### Ethnicity and health literacy

**Fig. 1:**
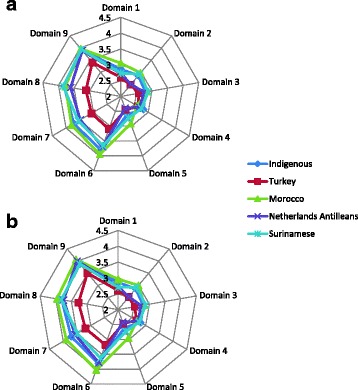
A summary of the health literacy scores of the different ethnic groups, divided over the nine domains. **a** presents the unadjusted means, and **b** shows the means adjusted for age and education

Multiple ANCOVAs with age and education as covariates revealed that there are significant differences in the means of the groups in domain two *(p < 0.01)*, domain six *(p < 0.01)*, domain seven *(p < 0.01)*, domain eight *(p < 0.01)* and domain nine *(p = 0.02)*. After the ANCOVA analyses are performed, the error variances are checked on normality. Pairwise comparisons of the adjusted means show that only the Turkish respondents significantly differ from the indigenous population (domain two *(p < 0.01)*, domain six *(p < 0.01)*, domain seven *(p < 0.01)*, domain eight *(p < 0.01)* and domain nine *(p < 0.01))*. These comparisons are calculated with the Tukey HSD test [31]. All results obtained from these analyses are shown in Table 2. The table shows consecutively the ANCOVA results for the unadjusted and the adjusted model, the results of the different ethnic groups compared to the indigenous population with the Tukey HSD test, and subsequently the adjusted means. The adjusted means are obtained through linear prediction. Figure 1 (a, b) summarizes the health literacy scores of the different ethnic groups, divided over the nine domains. Figure 1a presents the unadjusted means, and Fig. 1b shows the means adjusted for age and education.

**Table 3:** Models analyzing the relation of GP-visits with ethnicity and health literacy

Domain	Ethnicity			Health literacy		
	*B (SD)*	*IRR*	*p*	*B (SD)*	*IRR*	*p*
2 Sufficient information	**0.293 (0.14)**	**1.34**	**0.044**	−0.033 (0.12)	0.97	0.776
6 Active engagement	0.241 (0.15)	1.27	0.100	−0.142 (0.08)	0.87	0.064
7 Navigation	0.248 (0.15)	1.28	0.092	**−0.222 (0.08)**	**0.80**	**0.007**
8 Finding information	0.230 (0.15)	1.26	0.116	**−0.255 (0.08)**	**0.77**	**0.001**
9 Read and understand	0.245 (0.14)	1.28	0.092	**−0.191 (0.08)**	**0.83**	**0.024**

Significant results are printed in bold

### Ethnicity and GP visits

Negative binomial regression analysis shows that there is a significant association between ethnicity and GP visits, when adjusted for age, education and gender *(B = 0.282, p = 0.049)*. The calculated incidence rate ratio (IRR) of 1.33 clarifies that ethnic minority groups have 1.33 times as much, or 33% more, GP visits compared to the indigenous population. In the unadjusted model, this association is not significant *(B = -0.064, IRR = 0.94, p = 0.646)*.

### Health literacy as an explaining mechanism

To answer the third research question, health literacy domains are added in the model. Only the domains where ethnic minority groups significantly differed from the indigenous population in the analyses of the first research question were added. Table 3 shows that in all health literacy domains except domain 2, the association between ethnicity and GP visits is no longer significant. By contrast, in domain seven, eight and nine the association between health literacy and GP visits is significant. This reveals that health literacy domains seven (navigation), eight (finding information) and nine (read and understand information) are better predictors of GP visits than ethnicity.

Thereafter, interactions between ethnicity and health literacy domains are added to the regression model to see whether the effect of health literacy on GP visits significantly differs between the ethnic minority groups and the indigenous population. None of the interactions are significant, in respectively domain two *(p = 0.701)*, domain six *(p = 0.957)*, domain seven *(p = 0.581)*, domain eight *(p = 0.116)*, domain nine *(p = 0.209)*. This means that people with the same health literacy score, but a different ethnicity, have an equal frequency of GP visits.

## Discussion

The aims of this study were to acquire insight into the level of health literacy of ethnic minorities in the Netherlands and to examine whether the relationship between ethnicity and GP visits can (partly) be explained by health literacy. Hereby we focused on a range of skills covering several health literacy domains instead of basic reading and writing skills (‘functional health literacy’) only. These domains were measured with the Health Literacy Questionnaire (HLQ).

### Level of health literacy of ethnic minorities in the Netherlands

In general, there were few differences in health literacy between the indigenous Dutch population and the ethnic minority groups in this study. Differences were only present in the Turkish population and exclusively in the following domains: two (*Having sufficient information)*, six (*Active engagement with health care provider)*, seven *(Navigating the health care system)*, eight *(Ability to find health information)*, and nine *(Reading and understanding health information)*. On these domains, the Turkish respondents scored lower. There were no differences with regard to health literacy between the indigenous respondents and the Moroccan, Surinamese and Netherlands Antillean groups.

The differences in the level of health literacy between the ethnic minorities and the indigenous population were smaller than expected from previous, international, research ([11];[25]). This might be due to the small sample size of our study, as well as possible selection bias. Since the study sample was drawn from a panel with written questionnaires, individuals with insufficient language skills and consequently possibly lower health literacy are not included.

### Relationship between ethnicity, GP visits and health literacy

With respect to health care use, our results confirm that ethnic minorities in the Netherlands visit the GP more often [33], on average 33% more compared to the indigenous population. This higher frequency of GP visits of ethnic minorities is partly explained by health literacy. Three domains (the ability to navigate, the ability to find information, and the ability to read and understand information) are most important for explaining differences in GP visits. In fact, they are more important than ethnicity itself. People with the same health literacy score on these domains, but a different ethnicity, have an equal frequency of GP visits.

Our findings correspond with another study in the Netherlands in which health literacy was examined as a possible predictor of GP visits [34]. In that study, the conceptualization of health literacy in terms of functional, communicative and critical skills was used [20]. Functional health literacy was found to be the only set of skills that significantly predicted the frequency of GP visits [34]. In line with Nutbeams’ definition, the domains eight (finding information) and nine (read and understand information) in the HLQ are equivalent to functional health literacy. The ability to navigate is closely linked to these skills. Therefore also in this study, functional health literacy seems to be the most important predictor of the number of GP visits, regardless of ethnicity, and even in a sample of respondents who volunteered to participate in a panel that makes use of questionnaires.

Although differences in health literacy levels in this study are only seen in the Turkish population, Beauchamp and colleagues have found in Australia that the level of health literacy is lower in ethnic minority populations, and she regards language as a leading issue herein [3]. However, the interest towards critical and interactive health literacy increases [8, 30]. Van der Heide et al. [34], for example, demonstrated that especially communicative skills are important for self-management behaviour. The fact that different domains of health literacy impact different kind of behaviours warrants the use of multidimensional health literacy measurement instruments.

There are several strengths and limitations to this study. The biggest strength is that so far no study has been done concerning the health literacy of ethnic minorities in the Netherlands. The second strength is the fact that in this study health literacy is conceptualized and measured in a multidimensional way, using the Health Literacy Questionnaire [21]. Another strength is that the answers on questions about health literacy are self-reported. The subjectivity of these answers reveal what is important or problematic from an individual’s own perspective.

The major limitations are the small sample size and the low response rate of this study. However, the percentage of ethnic minorities in our sample (9.1%) is comparable to the percentage in the Dutch population (11.7%) [6]. Furthermore, the data are obtained from the Dutch Health Care Consumer Panel. People who participate in a panel are expected to have a higher level of (health) literacy compared to the general population. This selection bias might lead to an overestimation of the level of health literacy of the respondents in our study, also in the ethnic minority subsample. For this reason, further research on ethnic minorities’ health literacy is needed using different methods of data collection than panel survey research. Another limitation is that we could not control for possible confounders such as job and income. However, education is regarded as a good proxy for these social economic factors. In future research, these factors should be included. Lastly, the data are measured cross-sectional and therefore might include recall bias concerning the amount of GP visits. However, both limitations are equal in both the indigenous population and the ethnic minority populations.

The findings that some health literacy domains are better predictors of GP visits than ethnicity need further attention. Important to know is how these domains impact health care use. Furthermore, future research should use a different design, which is more inclusive to people with lower (health) literacy. Lastly, measures of health care use, like GP visits, should be measured with the information from health care insurance companies or the administration of GPs. In this way, recall bias can be avoided for more adequate research.

## Conclusions

In conclusion, in the Netherlands differences in the level of health literacy were found between the Turkish ethnic minority population and the indigenous population. There were no differences with people from other ethnic minorities. This study also found an association between ethnicity and GP visits. The ability to navigate the health care system, to find information and to read and understand health information partly explain the association between ethnicity and GP visits. Further research is recommended to understand the pathways through which health literacy impacts health care use.
